# Clinical characteristics of hypertrophic olivary degeneration following brainstem or cerebellar hemorrhage

**DOI:** 10.1038/s41598-025-14912-1

**Published:** 2025-08-25

**Authors:** Lin Lin, Xiaofeng Ye, Jianming Zheng

**Affiliations:** 1 Department of Neurology, Mindong Hospital Affiliated to Fujian Medical University, Heshan Road No.89, Fu’an, 355000 Fujian Province China; 2 School of Clinical Medicine, Fujian Medical University, Fuzhou, 350122 China

**Keywords:** Inferior olivary nucleus degeneration, Brainstem hemorrhage, Cerebellar hemorrhage, Prognosis, Neuroscience, Diseases, Health care, Medical research, Neurology, Signs and symptoms

## Abstract

Patients who have experienced bleeding in the posterior circulation of the brain often develop Hypertrophic Olivary Degeneration (HOD). This condition can lead to new neurological problems several months after the initial hemorrhage, potentially worsening the overall outcome for these patients. However, its pathogenesis and prognosis remain inconclusive. The research included 214 patients diagnosed with brainstem or cerebellar hemorrhage, of which 36 developed secondary HOD. The study aimed to analyze the clinical data of these patients, investigate the risk factors associated with HOD development, and evaluate the prognosis for those affected. (1) No significant differences in common cerebrovascular risk factors, such as hypertension and diabetes, were observed between the HOD and non-HOD groups among patients with lesions involving the Guillain-Mollaret triangle (GMT) (*P* > 0.05). (2) The site of hemorrhage was significantly correlated with the location of HOD (*P* < 0.05). (3) A significant association was found between the primary lesion’s site and the interval before HOD onset (*P* < 0.05). (4) Patients in the HOD group showed poorer functional outcomes, reflected by higher mRS scores (Z =  −2.859, *P* = 0.004) and lower ADL scores (Z =  −2.859, *P* = 0.004). Among patients with brainstem or cerebellar hemorrhage, all individuals with HOD had lesions involving the GMT. A significant correlation was identified between the site of hemorrhage and the location of HOD. Cerebellar hemorrhage cases were associated with shorter intervals before HOD onset, and HOD was linked to significantly worse functional outcomes.

## Introduction

Hypertrophic Olivary Degeneration (HOD) is a rare neurological condition identified in certain patients following brainstem or cerebellar hemorrhage. These patients often develop new neurological deficits several months after their primary condition stabilizes. A distinctive imaging feature of HOD is the presence of abnormal signals in the region of the inferior olivary nucleus (ION) on T2-weighted imaging (T2WI)^1^. HOD arises due to trans-synaptic degeneration caused by disruption of afferent inputs to the ION^2^. Its clinical manifestations include palatal tremor, oculopalatal tremor, cerebellar ataxia, and various involuntary movements.

The pathological hallmarks of HOD, described by Goto et al. in 1981^3^, include neuronal cytoplasmic vacuolation and astrocytic hypertrophy, which are closely associated with its imaging features. Current studies suggest that HOD is predominantly linked to damage involving the Guillain-Mollaret triangle (GMT), a neural pathway comprising the red nucleus in the midbrain, the dentate nucleus in the cerebellum, and the ION in the medulla. Lesions within the GMT—caused by cerebrovascular disease, tumors, toxicity, or posterior fossa surgery—are believed to trigger HOD^4^.

Clinically, HOD often presents with an insidious or subacute onset, leading to frequent misdiagnoses or missed diagnoses. While case reports and small case series dominate the literature, comprehensive studies exploring the epidemiology, pathogenesis, and prognosis of HOD remain scarce. Furthermore, there is a lack of early warning indicators and effective therapeutic strategies, underscoring the need for more extensive research.

This study retrospectively analyzed 214 cases of brainstem or cerebellar hemorrhage diagnosed at Ningde Municipal Mindong Hospital between January 2010 and January 2021. Among these, 36 patients were identified with imaging-confirmed HOD during follow-up cranial Magnetic Resonance Imaging (MRI). By comparing clinical data between patients with and without secondary HOD, we aim to determine the incidence of HOD, identify associated risk factors, and assess the clinical characteristics and outcomes of HOD. This study seeks to improve clinicians’ understanding of HOD and provide a foundation for its early diagnosis and effective management.

## Methods

### Study design

**Fig. 1:**
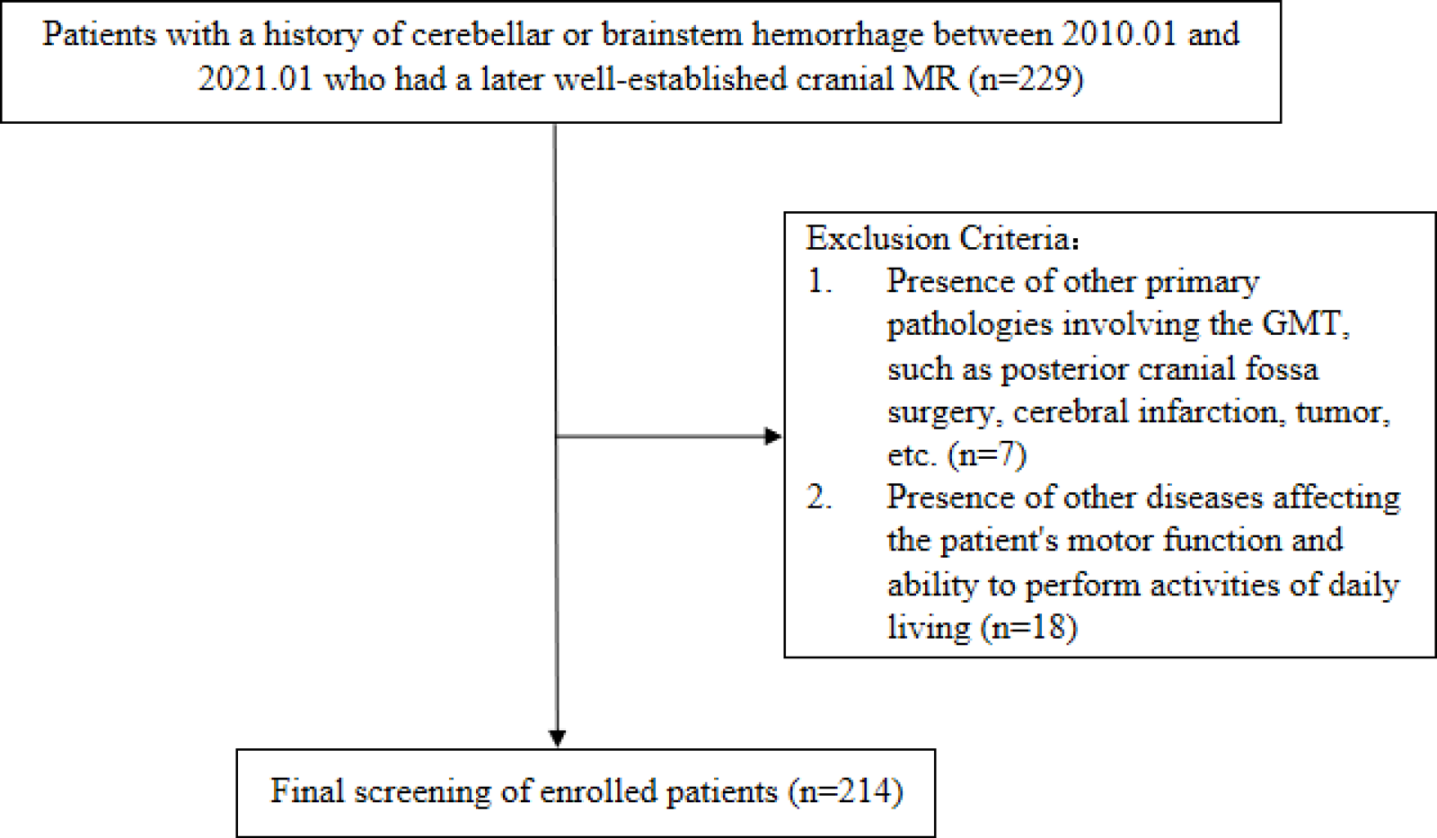
Inclusion and exclusion criteria.

Patients with brainstem or cerebellar hemorrhage diagnosed by cranial CT or cranial MRI at Mindong Hospital in Ningde City between January 2010 and January 2021 were collected and followed up.The inclusion and exclusion criteria are shown in Fig. 1.

#### Inclusion criteria

1. Age ≥ 18 years;
2. A confirmed diagnosis of brainstem or cerebellar hemorrhage by cranial CT or MRI;
3. Completion of follow-up cranial MRI after the hemorrhage

#### Exclusion criteria

1. Presence of other primary lesions involving the GMT (e.g., posterior fossa surgery, cerebral infarction, tumors);
2. Presence of other conditions affecting motor function or activities of daily living.

#### Grouping methods

Patients meeting the above inclusion and exclusion criteria were divided into three groups based on the involvement of GMT and the presence of HOD imaging features on follow-up cranial MRI:

- **Group A:** Patients with hemorrhagic lesions involving the GMT who exhibited HOD on follow-up MRI.
- **Group B:** Patients with hemorrhagic lesions involving the GMT but without HOD on follow-up MRI.
- **Group C:** Patients with hemorrhagic lesions not involving the GMT.

**Fig. 2:**
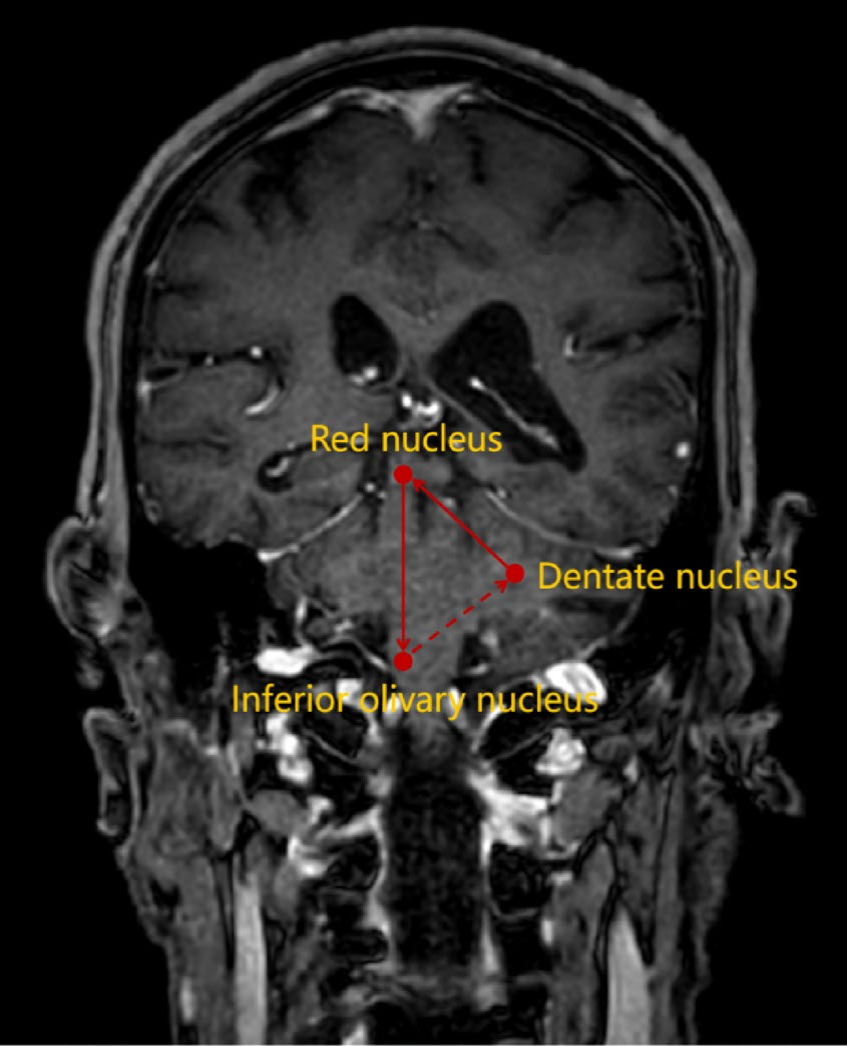
The anatomical location of the GMT on coronal cranial MRI.The GMT consists of the contralateral dentate nucleus in the cerebellum, the ipsilateral ION in the medulla oblongata, and the ipsilateral red nucleus at the level of the midbrain.

**Fig. 3:**
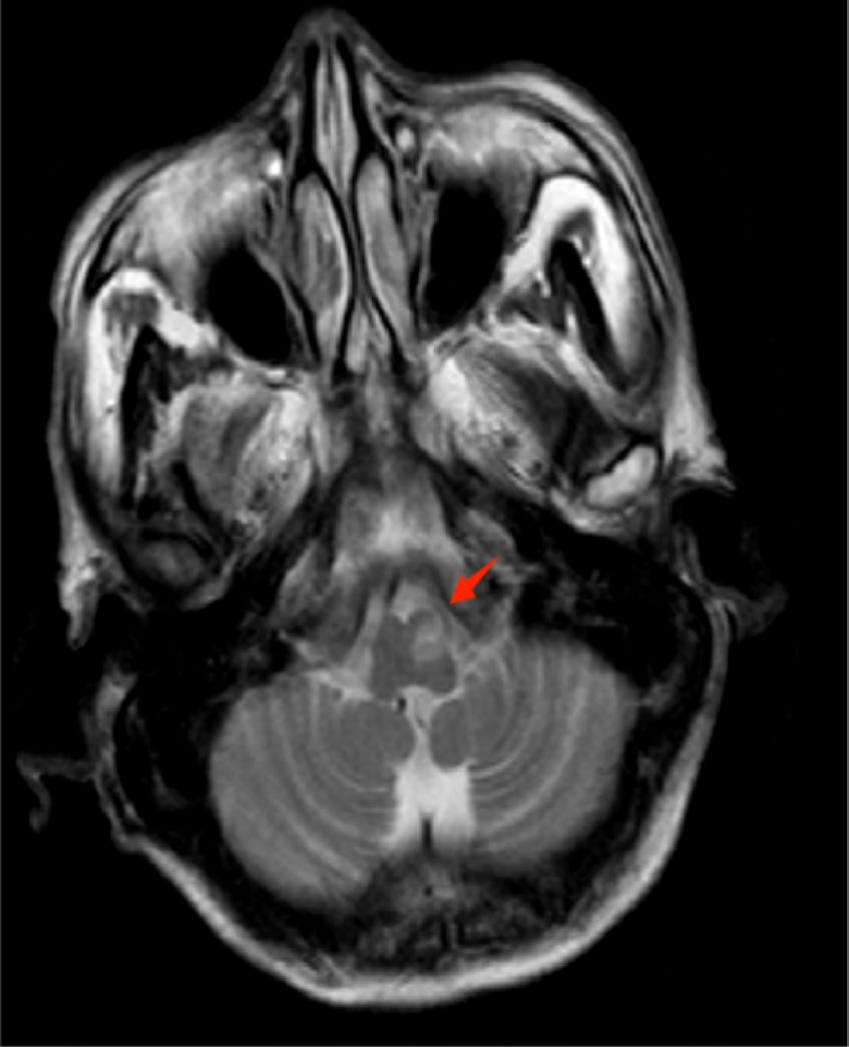
Typical imaging features of HOD. The abnormal signal in the region of ION located in ventrolateral medulla oblongata, with slightly high or high signal on T2WI and isointense or low signal on T1WI.

**Fig. 4:**
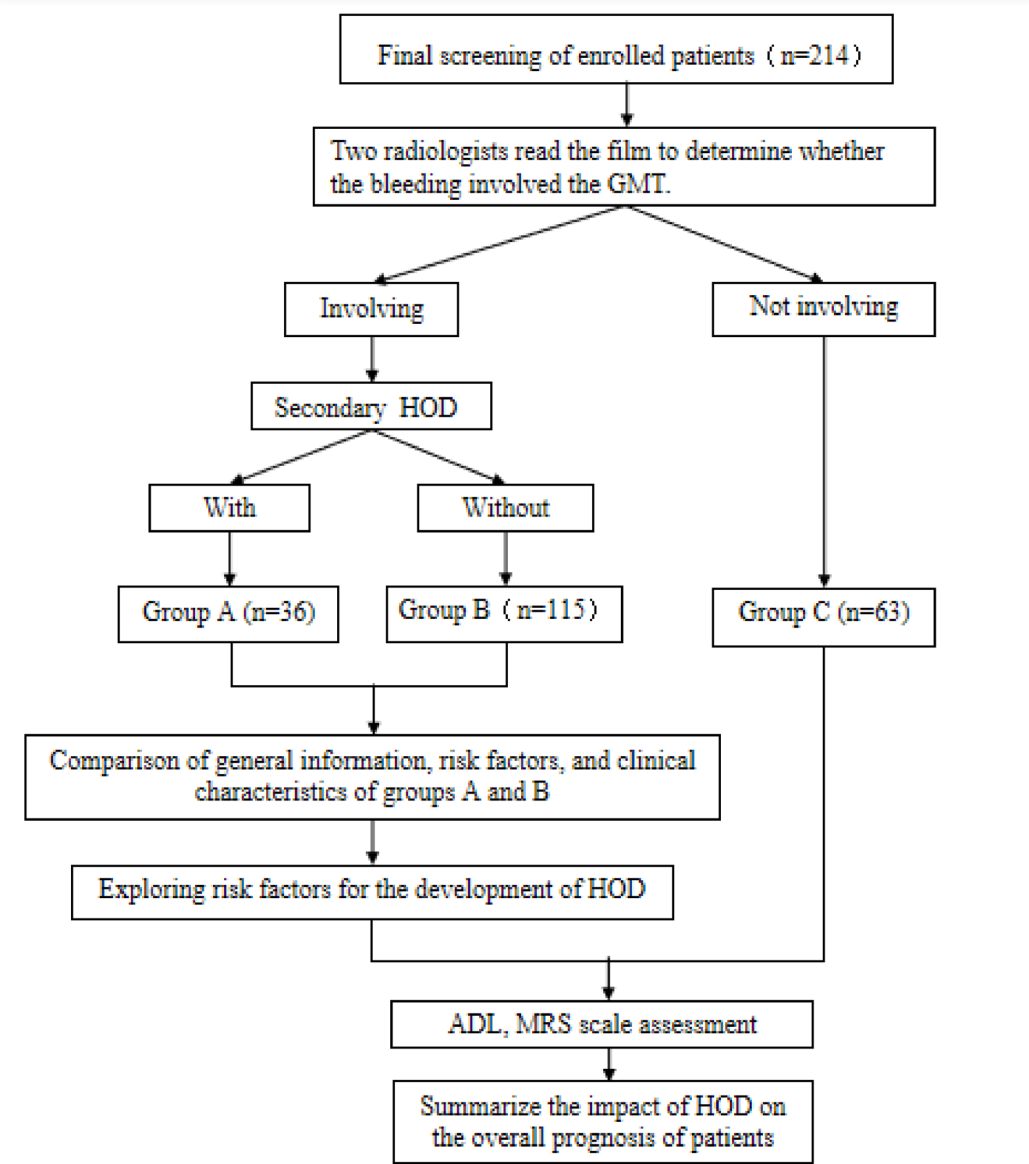
Flow chart.

All patients were independently evaluated by two radiologists to determine whether the hemorrhagic lesions involved the GMT and to calculate the volume of hemorrhage. The anatomical location of the GMT on coronal MRI is illustrated in Fig. 2. Follow-up imaging was independently reviewed by two experienced neurologists to confirm the presence of HOD. The characteristic imaging features of HOD are shown in Fig. 3. Communication or discussion between the radiologists and neurologists was strictly prohibited. A detailed study workflow is presented in Fig. 4.

### Data collection

Baseline data of patients with brainstem or cerebellar hemorrhage were collected, including vascular risk factors (hypertension, diabetes, dyslipidemia, hyperuricemia, smoking history, alcohol consumption history), clinical characteristics (hemorrhage volume, maximum systolic and diastolic blood pressure during hospitalization, hemorrhage location, HOD onset location and timing, clinical symptoms in HOD patients), and prognostic outcomes.

Hemorrhage volume was calculated using the ellipsoid formula proposed by Kothari:

V(hemorrhage volume) = A × B × C × 1/2,where A represents the maximum diameter of the hematoma on the largest axial slice, B represents the maximum perpendicular diameter on the same slice, and C equals the number of slices with hematoma multiplied by the slice thickness. The average of two independent measurements by radiologists was taken as the final result. Prognosis was assessed using the Modified Rankin Scale (MRS) and the Activities of Daily Living (ADL) scale.

### Statistical analysis

All statistical analyses were performed using SPSS version 26.0.

#### Exploration of risk factors

Descriptive statistics were used to summarize demographic and clinical data of Groups A and B, including hemorrhage volume. Statistical comparisons included:

- **t-tests** for age, maximum systolic blood pressure, maximum diastolic blood pressure, and LDL levels;
- **Mann–Whitney U tests** for hemorrhage volume, fasting blood glucose, HDL, triglycerides (TG), and uric acid;
- **Chi-square tests** for categorical variables such as sex, hemorrhage location, hypertension, diabetes, smoking history, and alcohol consumption history.

#### Analysis of clinical characteristics

- **Fisher’s exact test** was used to evaluate the relationship between hemorrhage location and HOD incidence, as well as the correlation between hemorrhage location and HOD site.
- **Spearman’s rank correlation** was employed to analyze the association between hemorrhage volume, the primary lesion site, and the interval to HOD onset.

#### Prognostic evaluation

- Differences in follow-up intervals, MRS scores, and ADL scores among Groups A, B, and C were analyzed using the Mann–Whitney U test.
- Differences in loss-to-follow-up rates were assessed using the Chi-square test.
- Mortality rates among the three groups were compared using the Chi-square trend test.
- Spearman’s rank correlation was used to examine the relationship between unilateral or bilateral HOD and MRS scores.

A significance level of *P* < 0.05 *P* < 0.05 *P* < 0.05 was set for all statistical tests.

## Results

### Baseline characteristics and clinical features

#### Baseline characteristics

Between January 2010 and January 2021, a total of 229 patients with brainstem or cerebellar hemorrhage were diagnosed via cranial CT or MRI at Ningde Mindong Hospital. After applying the inclusion and exclusion criteria, 214 patients were included in the final analysis. These patients were divided into three groups according to the classification criteria: Group A (36 patients), Group B (115 patients), and Group C (63 patients).

**Fig. 5:**
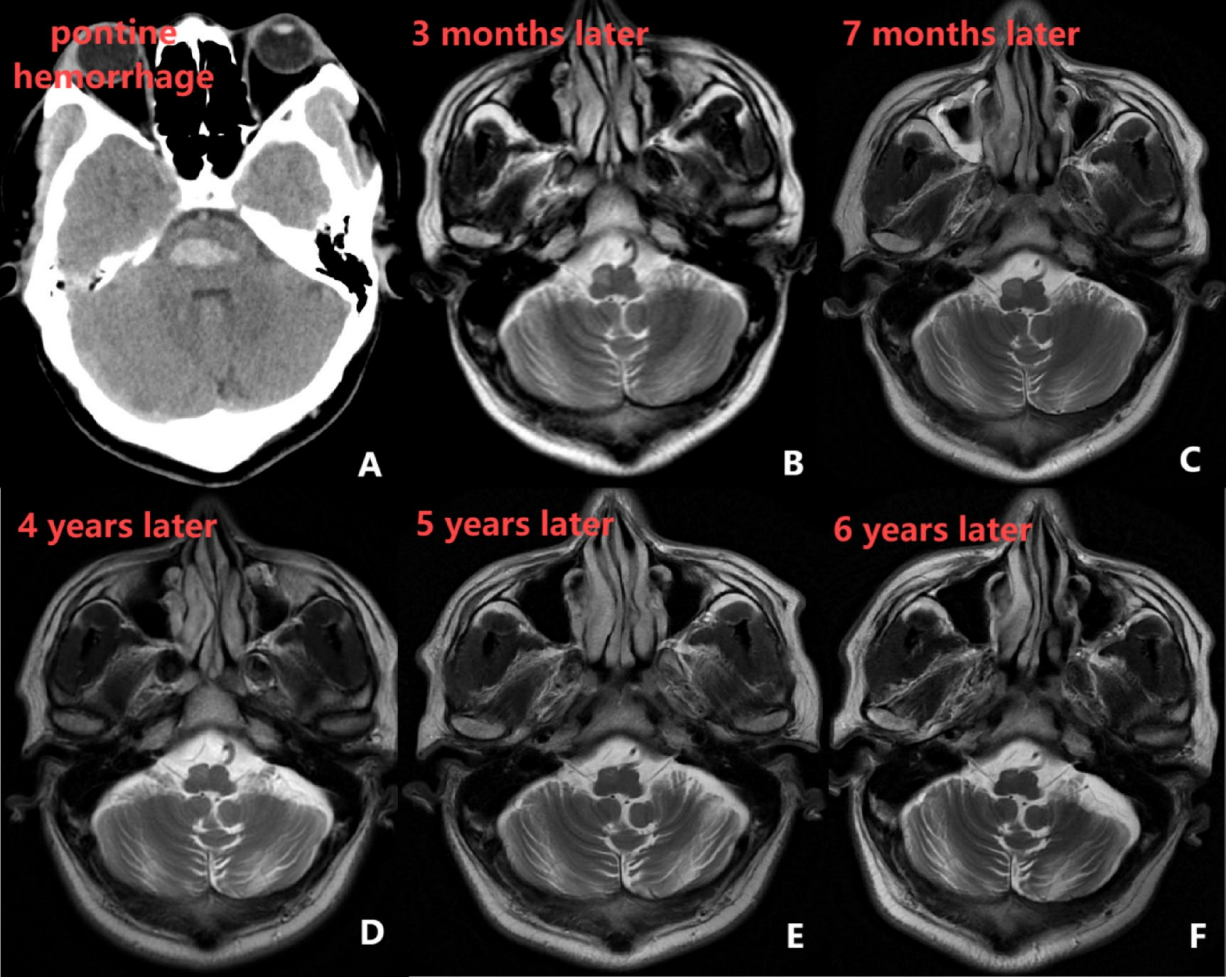
These MR image series depict the HOD dynamic process of patient 12 in Table 1 who had pontine hemorrhage (**A**).Within the first 3 months of insult, the ION has increased signal intensity on T2WI without hypertrophy (**B**); After 7 months,the ION showed hyperintensity and hypertrophy signal (**C**). Hypertrophy regresses gradually and only hyperintensity is seen 4 years later. Hyperintensity persists indefinitely. Over time, the olives undergoes atrophy (**D**–**F**).

All patients with HOD had primary hemorrhagic lesions involving the GMT. Among patients with brainstem or cerebellar hemorrhage, the prevalence of HOD was 16.82%. Figure 5 illustrates the dynamic signal changes of HOD on cranial MRI for a representative patient (case number 12 in Table 1).

**Table 1:** Clinical characteristics of 36 patients with secondary HOD following brainstem or cerebellar hemorrhage.

No	Age (years)	Gender	Primary Lesion	Hemorrhage volume (mL)		Onset (days)	HOD location	Clinical manifestations	Outcome	MRS	ADL
1	77	Male	Brainstem	0.81		121	Left	None	Death	−	−
2	57	Male	Brainstem	1.37		91	Right	Dizziness,Tremors	Survival	1	100
3	62	Male	Brainstem	0.964		1441	Left	None	Losttofollow − up	−	−
4	63	Female	Brainstem	3.62		115	Right	Dizziness,SlurredSpeech	Survival	4	35
5	61	Female	Brainstem	3.156		331	Left	None	Losttofollow − up	−	−
6	44	Female	Brainstem	8.348		162	Bilateral	MotorDysfunction,Dizziness,SlurredSpeech,Ataxia	Survival	4	30
7	41	Female	Brainstem	2.296		1066	Right	Dizziness,OcularMovementDisorder,Nystagmus,Ataxia	Survival	2	100
8	49	Male	Brainstem	2.06		633	Right	OcularMovementDisorder,Nystagmus,SlurredSpeech	Survival	3	95
9	51	Male	Brainstem	2.857		75	Left	None	Death	−	−
10	52	Male	Brainstem	4.1764		201	Bilateral	None	Losttofollow − up	−	−
11	43	Male	Brainstem	7.722		41	Bilateral	None	Death	−	−
12	32	Male	Brainstem	2.115		81	Right	None	Losttofollow − up	−	−
13	44	Male	Brainstem	0.507		143	Right	Nystagmus,Tremors	Survival	3	100
14	59	Female	Brainstem	1.136		496	Right	None	Losttofollow − up	−	−
15	57	Male	Brainstem	1.662		18	Bilateral	Tremors,SlowResponse	Survival	4	70
16	58	Female	Brainstem	0.844		13	Right	None	Losttofollow − up	−	−
17	52	Female	Brainstem	3.08		152	Bilateral	PoeticSpeech,InvoluntaryMovements	Survival	4	45
18	47	Male	Brainstem	1.34		73	Left	PalatalMyoclonus,MotorDysfunction,Nystagmus,OcularMovementDisorder,SlurredSpeech,Ataxia,Tremors	Survival	4	40
19	66	Female	Brainstem	8.44		212	Bilateral	SlurredSpeech,MotorDysfunction,RestTremor,LimitedLeftEyeAbduction	Survival	4	20
20	73	Male	Cerebellum	3.33		157	Right	Dizziness,SlurredSpeech	Survival	3	40
21	71	Male	Cerebellum	10.947		29	Left	None	Survival	3	85
22	54	Female	Cerebellum	1.068		15	Bilateral	Dizziness	Survival	0	100
23	67	Female	Cerebellum	0.465		723	Right	Dizziness,MotorDysfunction	Survival	2	85
24	40	Male	Cerebellum	10.605		65	Right	None	Losttofollow − up	−	−
25	62	Male	Cerebellum	6.291		10	Left	None	Losttofollow − up	−	−
26	76	Female	Cerebellum	9.195		149	Left	Dizziness	Survival	5	100
27	70	Male	Cerebellum	1.762		707	Right	None	Losttofollow − up	−	−
28	69	Male	Cerebellum	0.9686		141	Left	None	Death	−	−
29	63	Female	Cerebellum	19.954		24	Left	InvoluntaryMovements,SlurredSpeech,Ataxia	Survival	3	75
30	82	Female	Cerebellum	4.8564		10	Right	None	Losttofollow − up	−	−
31	65	Male	Cerebellum	3.0625		5	Left	None	Losttofollow − up	−	−
32	65	Male	Cerebellum	1.3277		195	Left	Ataxia,InvoluntaryMovements	Survival	1	100
33	52	Male	Cerebellum	8.12		24	Right	Dizziness	Survival	2	100
34	71	Male	Cerebellum	0.906		14	Left	InvoluntaryMovements,Ataxia	Survival	3	85
35	50	Female	Cerebellum	2.697		88	Right	Ataxia,SlurredSpeech,Dizziness,Tremors	Survival	1	100
36	61	Female	Cerebellum	5.096		17	Left	MotorDysfunction,Tremors,Dizziness,SlurredSpeech	Survival	4	65

#### Clinical features

Among the 36 patients with secondary HOD, 4 died, 21 were followed up, and 11 were lost to follow-up. The relevant clinical symptoms of the patients who were followed up are summarized in Table 1.

### Exploration of factors associated with HOD onset

#### Common cerebrovascular risk factors and HOD

In both Groups A and B, the distribution of patients with brainstem hemorrhage and cerebellar hemorrhage was balanced, indicating no significant correlation between the location of the primary hemorrhagic focus and the onset of HOD. There were no statistically significant differences between Group A and Group B in terms of age, gender, hemorrhage volume, hemorrhage location, maximum systolic blood pressure, maximum diastolic blood pressure, smoking, alcohol consumption, or low density lipoprotein (LDL) levels, which are common risk factors for cerebrovascular diseases (*P* > 0.05 for all).

#### HOD location and hemorrhage site

##### Correlation between HOD location and hemorrhage site in brainstem hemorrhage patients

Among the 19 patients with brainstem hemorrhage:

- 3 patients with left-sided hemorrhage developed left-sided HOD
- 1 patient with left-sided hemorrhage developed bilateral HOD
- 7 patients with right-sided hemorrhage developed right-sided HOD
- 8 patients with bilateral brainstem hemorrhage:
  - 5 patients developed bilateral HOD
  - 2 patients developed left-sided HOD
  - 1 patients developed right-sided HOD

**Fig. 6:**
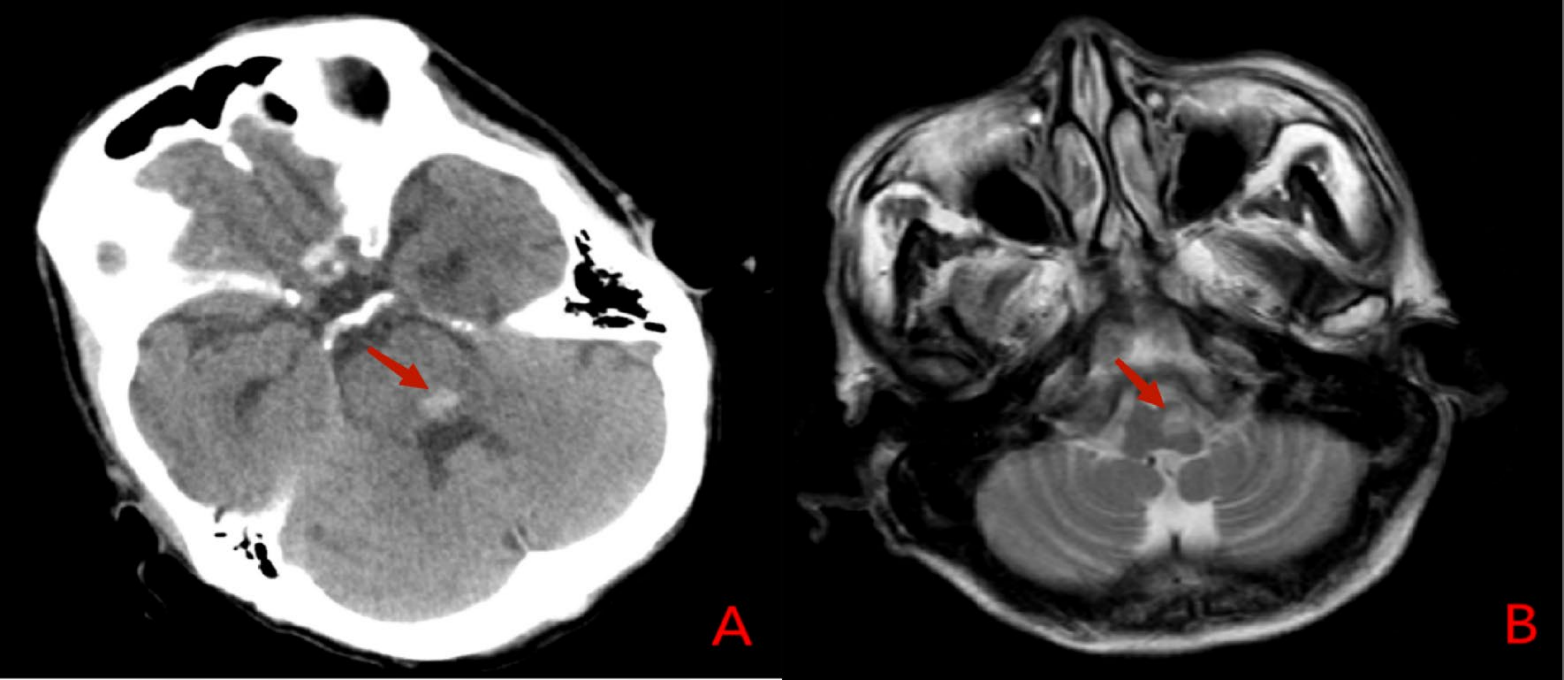
Case No.1, Head computed tomography (**A**) revealed pontine hemorrhage on the left side; T2WI (**B**) revealed hyperintensity on the ipsilateral ION 4 months after the insult.

**Fig. 7:**
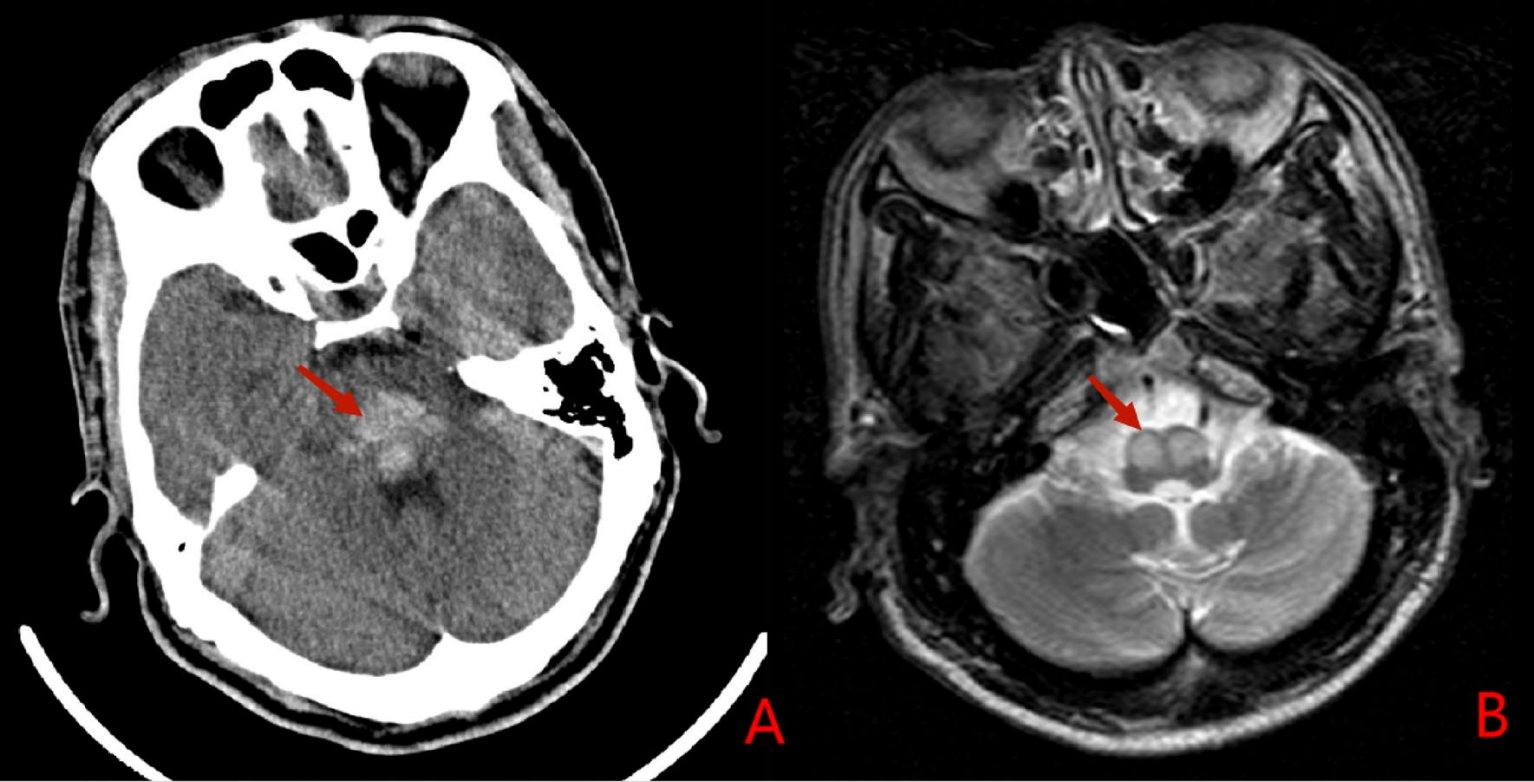
Case No.10, Head computed tomography (**A**) revealed pontine hemorrhage on both sides; T2WI (**B**) revealed hyperintensity on the both ION 7 months after the insult.

Fisher’s exact test was performed to assess the correlation between hemorrhage site and HOD location in brainstem hemorrhage patients, yielding χ^2^ = 16.136 (*P* < 0.001). A significant correlation was found between the site of brainstem hemorrhage and the location of HOD, with HOD most commonly occurring on the same side as the hemorrhage. Figures 6 and 7 show imaging changes in representative brainstem hemorrhage patients.

##### Correlation between HOD location and hemorrhage site in cerebellar hemorrhage patients

Among the 17 patients with cerebellar hemorrhage:

- 7 patients with left-sided cerebellar hemorrhage developed right-sided HOD,
- 9 patients with right-sided cerebellar hemorrhage developed left-sided HOD
- 1 patient with right-sided cerebellar hemorrhage developed bilateral HOD.

**Fig. 8:**
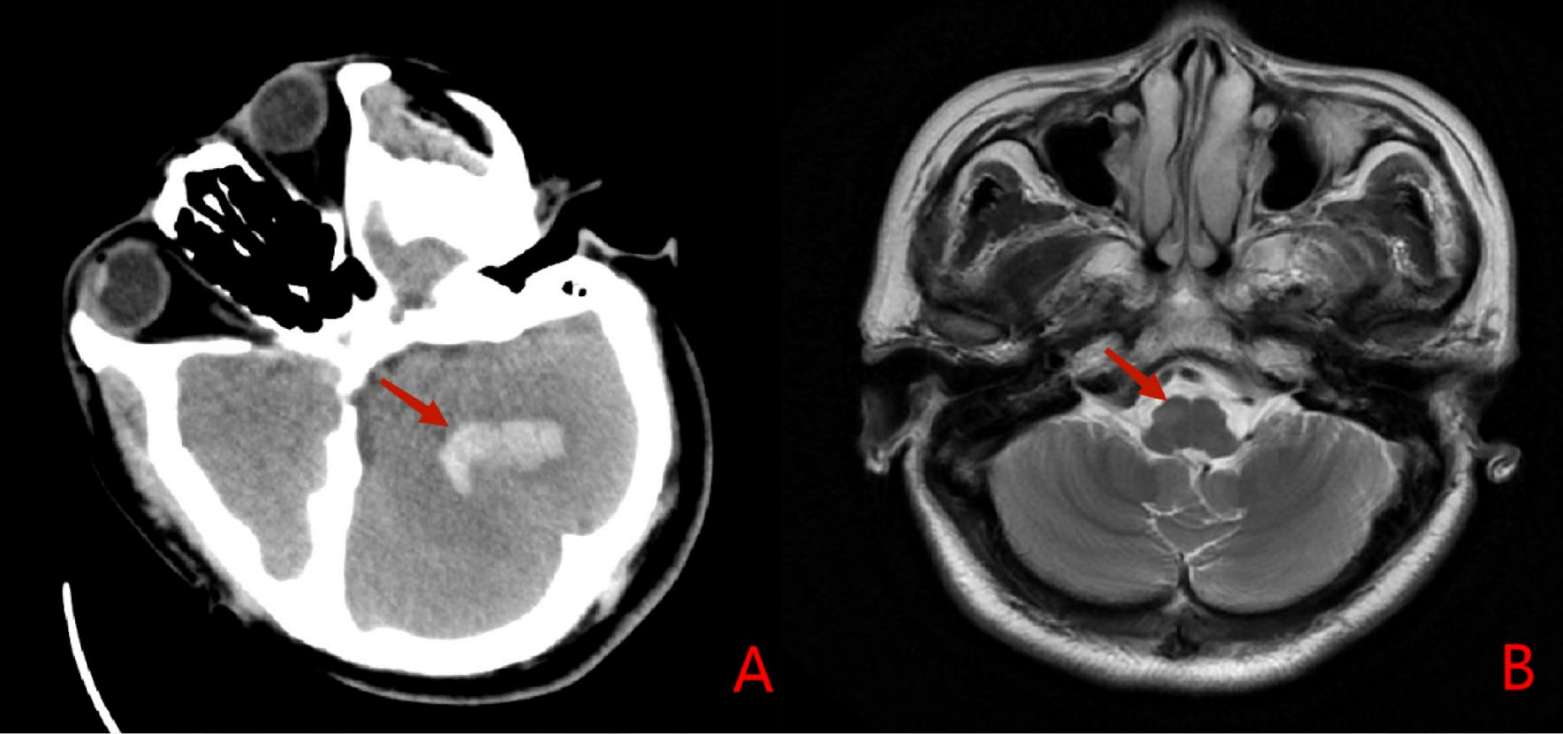
Case No.35, Head computed tomography (**A**) revealed cerebellar hemorrhage on the left side; T2WI (**B**) revealed hyperintensity on the contralateral ION 3 months after the insult.

Fisher’s exact test was performed to assess the correlation between hemorrhage site and HOD location in cerebellar hemorrhage patients, yielding χ^2^ = 17.601 (*P* < 0.001). A significant correlation was found between the site of cerebellar hemorrhage and the location of HOD, with HOD most commonly occurring on the opposite side of the hemorrhage. Figure 8 show imaging changes in representative cerebellar hemorrhage patients.

#### Interval between HOD onset and hemorrhage site

Among the 36 patients who developed HOD, the median interval from the diagnosis of cerebral hemorrhage to imaging confirmation of HOD was 143 days. Spearman correlation analysis between the site of the primary lesion and the interval to HOD onset revealed a correlation coefficient of −0.326 (*P* = 0.03, *P* < 0.05), indicating a weak but significant correlation between the primary lesion site and the delayed onset of HOD. Compared to patients with brainstem hemorrhage, those with cerebellar hemorrhage had a shorter interval to HOD onset.

#### Interval between HOD onset and hemorrhage volume

Spearman correlation analysis of hemorrhage volume and the time to HOD onset showed a correlation coefficient of −0.203 (*P* = 0.234, *P* > 0.05), indicating no significant correlation between hemorrhage volume and the interval to HOD onset.

### Prognosis of HOD patients

#### Follow-up of patients in ABC groups

Among the 214 enrolled patients with brainstem or cerebellar hemorrhage:

- Group A: 21 survivors, 4 deaths, 11 lost to follow-up
- Group B: 63 survivors, 12 deaths, 40 lost to follow-up
- Group C: 32 survivors, 8 deaths, 23 lost to follow-up

There were no significant differences in the interval between cerebral hemorrhage and follow-up, or in the loss to follow-up rates among the three groups (*P* > 0.05).

#### Prognosis of patients in ABC groups

After excluding lost-to-follow-up patients, prognosis data were available for 140 patients. Using the MRS and ADL scales to assess the prognosis of survivors, we found no significant difference in mortality rates among the three groups. However, there were significant differences in the MRS and ADL scores (*P* < 0.05 for both). The proportion of patients in Group A with an MRS score ≥4 was significantly higher than in the other two groups, and the median ADL score in Group A was lower than in Group B, suggesting a poor prognosis for patients who develop HOD.

#### Impact of unilateral or bilateral HOD on prognosis

Among the patients in Group A who were followed up, 16 had unilateral HOD and 5 had bilateral HOD. Spearman correlation analysis between unilateral/bilateral HOD and MRS and ADL scores showed a correlation coefficient of 0.267 (*P* = 0.242, *P* > 0.05) for MRS and −0.39 (*P* = 0.081, *P* > 0.05) for ADL, indicating no significant correlation between the presence of unilateral or bilateral HOD and the MRS or ADL scores.

## Discussion

Hypertrophic Olivary Degeneration (HOD) is a form of transsynaptic degeneration caused by lesions in the dentato-rubro-olivary pathway. It was first described by Oppenheim in 1887 during autopsy. In 1931, Guillain and Mollaret proposed that the onset of HOD was associated with damage to the dentato-rubro-olivary pathway and detailed its anatomical relationship. The dentate nucleus connects to the contralateral red nucleus through the superior cerebellar peduncle and the dentato-rubral tract, while the red nucleus connects to the ipsilateral ION via the central tegmental tract. The output fibers from the ION cross to the contralateral side, project through the contralateral inferior cerebellar peduncle to the cerebellar cortex, and then project back to the dentate nucleus, forming a triangular structure known as the Guillain-Mollaret triangle (GMT)^5^. Research indicates that HOD can be triggered by any damage to the GMT occurring superior to the ION^4^. A wide range of underlying conditions has been associated with this disorder, as documented in various case reports. While cerebrovascular diseases are the most frequently reported cause^6^, other etiologies include neoplasms, toxic exposures, complications from posterior fossa surgical procedures, encephalitis, neuro-Behçet’s disease, and Wilson’s disease. This diverse array of potential causes highlights the complexity of the condition and the importance of thorough diagnostic evaluation. The identification of HOD primarily depends on its distinctive clinical manifestations and imaging findings. Typically, patients exhibit symptoms such as palatal tremor, nystagmus, and cerebellar ataxia, which paradoxically appear as their initial neurological deficits improve. Magnetic Resonance Imaging (MRI) usually reveals an enlarged inferior olive with prolonged T2 signal intensity. However, these clinical and radiological features closely resemble the aftereffects of posterior circulation injuries, frequently resulting in diagnostic errors or delays. This similarity poses a significant challenge in accurately distinguishing HOD from other post-injury complications, potentially impacting timely and appropriate patient management. Further emphasizing the diagnostic challenges, a clinical study examining 12 cases of stroke-related HOD revealed that while all patients with GMT lesions exhibited imaging evidence of HOD, only half were correctly identified by clinicians^7^. The current body of research on HOD is largely limited to case reports and small-scale analyses, leaving a gap in comprehensive understanding of its prevalence, clinical characteristics, and long-term outcomes. To address this knowledge deficit, our study encompassed a larger cohort of 214 patients diagnosed with brainstem or cerebellar hemorrhages over an 11-year period from January 2010 to January 2021. Among these, 36 patients were diagnosed with secondary HOD. Our findings indicate that HOD is not an uncommon sequela in patients with cerebellar or brainstem hemorrhages. Moreover, we observed a correlation between the hemorrhage location and the site of HOD development. Notably, patients with cerebellar hemorrhages tended to develop HOD more rapidly compared to those with brainstem hemorrhages. Importantly, our results suggest that patients who develop secondary HOD generally face a less favorable prognosis.

Contrary to earlier perceptions of HOD as a rare condition ^8,9^, recent research suggests it is more prevalent in patients with posterior circulation injuries. Gautier JC et al.^2^ reported HOD development in 62.5–75% of stroke patients with midbrain tegmentum damage. Our study corroborates this finding, with 16.82% (36 out of 214) of patients with brainstem or cerebellar hemorrhage diagnosed with HOD. However, this percentage may be an overestimation due to the retrospective nature of the study and potential selection bias from including only patients who underwent follow-up MRI. While theoretically any patient with a primary lesion involving the GMT could develop HOD, it appears more common following hemorrhages than infarctions in the brainstem or cerebellum. The exact mechanism for this disparity remains unclear^10^, and our study specifically focused on hemorrhagic cases. These findings underscore the need for increased awareness and further research into HOD, particularly in the context of posterior circulation injuries.

The GMT is crucial for coordinating movement and maintaining posture. When this structure is compromised, HOD frequently occurs as a secondary effect^11^. The majority of research indicates that HOD’s development is directly linked to GMT damage^4,5^. A wide range of medical literature, primarily in the form of case studies, has documented various conditions that can result in HOD through GMT injury,including pontine haemorrhage^12^, cortical-basal ganglia degeneration^13^, and posterior fossa surgery^14^. This diverse array of potential causes underscores the vulnerability of the GMT and the consequent risk of HOD in a variety of neurological conditions and procedures. In our study, all patients with secondary HOD had lesions involving the GMT, further supporting the association between GMT injury and the development of HOD. However, some studies have reported that a portion of HOD patients show no obvious lesions within the GMT ^6,15,16^. In a large retrospective cohort study of 102 HOD patients, Carr CM et al.^15^ found that 76% of patients had bilateral lesions, but 44% had no visible lesions in the GMT. Similarly, Konno T et al. ^6^ observed the same pattern in a study of 95 HOD patients. Madhavan Aet al. ^16^ noted that nearly half of patients with HOD had no lesions in the GMT in 2021. These patients often present with progressive ataxia and palatal tremor syndrome, which may be sporadic or familial. In sporadic patients, MRI usually shows changes consistent with HOD, even when no structural abnormalities are found in the GMT, while familial cases may not show HOD. This suggests that other underlying mechanisms may contribute to the pathogenesis of HOD ^17^. Regarding risk factors for HOD, no conclusive evidence exists. In this study, factors such as age, hypertension, diabetes, hyperlipidemia, hyperuricemia, smoking, and alcohol consumption were evaluated but no significant association with HOD incidence was found. The relatively small number of HOD cases in this study may have limited the exploration of potential risk factors.

The clinical symptoms of HOD typically include palatal tremor, nystagmus, cerebellar ataxia, and other involuntary movements. Among the 36 HOD patients studied, the predominant symptoms were dizziness (30.56%), dysarthria (25%), and both limb tremor and ataxia (19.45%). Some studies suggest that palatal myoclonus is the most common clinical manifestation of HOD ^18^, though in this study, only 1 of the 21 patients who completed follow-up underwent electronic laryngoscopy, revealing palatal myoclonus. This result was likely influenced by the low follow-up rate and lack of cooperation for laryngoscopy. Palatal tremor in HOD patients is typically characterized by rhythmic contractions of the soft palate and pharyngeal muscles, resulting in involuntary movements at a frequency of 1.5–3 Hz, occasionally involving the facial, tongue, or laryngeal muscles^19,20^. However, the absence of palatal tremor does not rule out the diagnosis of HOD^21^. The onset of HOD symptoms is usually delayed, and palatal tremor can persist even after other symptoms subside. Rieder et al.^22^ proposed that this could be related to atrophy of the motor pathways caused by HOD. Dizziness, being a nonspecific symptom, may be related to damage to the cerebellum or vestibular nuclei, as well as nystagmus and other eye movement disorders. In brainstem hemorrhage patients, some lesions may involve the corticobulbar tract, causing unclear pronunciation of consonants, which leads to dysarthria. In cerebellar hemorrhage patients, ataxic dysarthria may be associated with dysarthria. Eye movement and limb activity disorders are often sequelae of brainstem and cerebellar hemorrhages but may be exacerbated following the development of HOD. HOD patients may experience various forms of nystagmus, causing visual problems and dizziness that affect daily functioning^23^. Holmes tremor, a low-frequency (< 4.5 Hz) and unilateral upper limb tremor^24^ often associated with HOD, was not observed in our study. Holmes tremor is characterized by a resting tremor that worsens with posture or intention, and disappears during sleep^25^. Remy et al.^26^ suggested that its occurrence is linked to damage to dopaminergic pathways and the cerebellar-thalamic/cerebellar-olivary systems.The retrospective nature of this study, combined with long follow-up periods and lost follow-up data, may have influenced the assessment of symptom progression.

MRI remains the gold standard for diagnosing HOD, as most patients lack clear clinical manifestations at diagnosis. Typical imaging findings include abnormal signals on T2WI in the region of the ION in the anterolateral medulla,along with contralateral cerebellar atrophy. Although this imaging feature is characteristic of HOD, it lacks specificity. Similar T2 hyperintensities can also be seen in conditions such as cerebral infarction, tumors, demyelinating diseases, infections, and Wernicke-Korsakoff syndrome.However, the diagnosis of HOD is confirmed when there is concomitant palatal tremor or primary lesions are found within GMT^10^. MRI changes in HOD follow a dynamic progression. A meta-analysis by Goyal et al.^4^ summarized the patterns of HOD changes on MRI as follows: (1) within the first six months post-injury, T2WI and proton density-weighted imaging (PDWI) typically show hyperintensities, without any enlargement of the inferior ION; (2) between six months and three years post-injury, hyperintensities persist on T2WI and PDWI, accompanied by hypertrophy of the ION; (3) after three years, the enlargement of the ION gradually resolves, while hyperintensities on T2WI and PDWI continue, followed by gradual atrophy of the olivary nucleus.In this study, the median time from the diagnosis of cerebral hemorrhage to MRI confirmation of HOD in 36 patients was 143 days, consistent with the described progression. Some researchers have proposed classifying HOD into three types based on the location of the lesion within GMT: (1) primary lesions involving the dentate nucleus or superior cerebellar peduncle lead to contralateral HOD; (2) lesions affecting the central tegmental tract result in ipsilateral HOD; (3) bilateral lesions in the aforementioned pathways result in bilateral HOD^27,28^. However, since there is no direct connection between the ION and the contralateral dentate nucleus, damage to the afferent fibers between these structures typically does not lead to HOD ^29^. In this study, an analysis of the hemorrhage site and the location of HOD revealed that brainstem hemorrhages were predominantly associated with HOD on the same side, while cerebellar hemorrhages were more often linked to contralateral HOD, reinforcing this observed pattern.However, among the 36 patients, two cases of unilateral hemorrhage resulted in bilateral HOD, and three cases of bilateral hemorrhage resulted in unilateral HOD, a phenomenon also reported in the literature ^30^.This could be attributed to lesions causing bilateral HOD that surpass the resolution limits of clinical MRI or to the delayed appearance of certain HOD changes^31^. Furthermore, cohort studies have documented two cases where initial MRI showed unilateral enlargement of the ION, which later progressed to bilateral HOD during follow-up^15^. In our study, unilateral HOD was more common, accounting for 80.56% of cases, which is consistent with most case reports and small-sample analyses^10,32^. However, two retrospective cohort studies from 2015 and 2016 found that bilateral HOD was more prevalent than unilateral HOD, with the proportion of bilateral cases reaching 76% and 56%, respectively. Notably, most unilateral HOD cases had distinct lesions within GMT, whereas bilateral HOD was more frequently observed in patients without distinct lesions in the GMT. These findings suggest that the underlying mechanisms of unilateral HOD and certain cases of bilateral HOD may differ^6,15^.

The onset of HOD typically occurs within a few months after the initial hemorrhagic event, and neuroimaging plays a pivotal role in its diagnosis. However, due to its delayed manifestation, early imaging may fail to reveal characteristic signs of HOD. In this study, a weak correlation was observed between the latency to HOD onset and the location of the primary hemorrhage. Specifically, patients with cerebellar hemorrhage exhibited a shorter interval of HOD development compared to those with brainstem hemorrhage. A review of the literature suggests that this heterogeneity may be attributed to more direct anatomical connections between the cerebellum and the inferior olivary nucleus, which likely facilitate more rapid trans-synaptic transmission of pathological signals^33^. This close structural and functional coupling is thought to accelerate the degenerative cascade, thereby reducing the latency to HOD onset. Among the 17 patients in this study with HOD secondary to cerebellar hemorrhage, the hemorrhagic lesions directly involved the dentate nucleus. Neural signals could thus be transmitted via the superior cerebellar peduncle to the contralateral red nucleus, and subsequently affect the ipsilateral inferior olivary nucleus through the central tegmental tract. This direct neural pathway may expedite the degenerative process by promoting paracrine trans-synaptic degeneration within the inferior olivary nucleus^34,35^. In contrast, brainstem hemorrhages primarily involve the central tegmental tract or red nucleus, where signal propagation requires crossing fibers and thus follows a longer, less direct route, potentially leading to a delayed onset of degeneration^17,36^. In this cohort, 19 patients with brainstem hemorrhage exhibited lesions localized to the pons, involving the GMT. Given the homogeneity of lesion location, further subclassification was not performed. A 2020 meta-analysis of 13 patients with brainstem infarcts—including nine with pontine involvement—reported no significant correlation between infarct location within the brainstem and the timing of HOD onset. However, that study did not include patients with cerebellar hemorrhage, which may account for differences in findings. As HOD can be clinically silent in its early stages, the timing of follow-up imaging and clinical evaluations is critical for detecting its onset. Accurate detection hinges on appropriately timed surveillance, as supported by numerous studies ^10,14,37,38^. Because this was a retrospective study, the recorded time to HOD onset may have been influenced by variations in the duration of follow-up, potentially introducing selection bias. Therefore, systematic and prospective monitoring of patients with brainstem or cerebellar hemorrhage would be valuable for precisely capturing the evolution of HOD and enhancing the robustness of future studies.

The prognosis of HOD remains poorly understood, with limited studies available on the subject. There is no consensus on the exact prognosis of HOD patients. Most studies indicate a poor prognosis for these patients, with symptomatic treatments yielding limited effectiveness and little to no relief of symptoms in most reported cases^19^. In the present study, the overall prognosis of 116 surviving patients was assessed using the Modified Rankin Scale (MRS) and Activities of Daily Living (ADL) scale. Significant differences were observed in MRS and ADL scores across the three groups. Compared to Group B, patients in Group A had a higher proportion of MRS scores ≥ 4, and a lower median ADL score. No significant differences in MRS and ADL scores were observed between Groups B and C. These findings suggest that secondary HOD patients experience a higher incidence of severe disability and greater impairment in daily living activities, which contributes to a poorer overall prognosis. This result aligns with the findings from most case reports.

Currently, the treatment for HOD primarily focuses on symptom management. Shaikh et al.^39^ proposed that medications enhancing GABAergic inhibition (e.g., clonazepam, alprazolam, or topiramate), reducing glutamatergic excitability (e.g., memantine or topiramate), or decreasing electro-coupling (e.g., quinine or mexazolam) may alleviate HOD symptoms. Additionally, there have been reports of successful treatment of involuntary facial and pharyngeal muscle movements through botulinum toxin injections into the orbicularis oris^40^. In recent years, transcranial direct current stimulation (tDCS)^41^ and transcranial magnetic stimulation (TMS)^42^ have also been proven to be effective and are increasingly used in the treatment of HOD. However, some studies suggest that HOD is a self-limiting condition,and excessive intervention may not be necessary^43^. In 2016, Takuya et al.^6^ reported that symptoms in unilateral HOD patients tended to improve over time, whereas those with bilateral HOD may experience disease progression. However, in this study, no correlation was found between the laterality of HOD and prognosis,which may be attributed to the limited number of bilateral HOD cases included and the extended follow-up duration in this cohort.

Given the retrospective nature of this study, selection bias may have influenced the results, particularly due to incomplete follow-up data and variations in patient behavior. Additionally, because some patients had long follow-up intervals, and a high dropout rate, it was difficult to accurately assess symptom changes over time.

## Conclusion

In conclusion, our study demonstrates that HOD is not uncommon in patients with brainstem or cerebellar hemorrhages and that the location of hemorrhage is correlated with the development of HOD. Secondary HOD is associated with a poor prognosis. This suggests that clinicians should regularly screen for HOD in patients with brainstem or cerebellar hemorrhages, particularly those with hemorrhagic lesions involving the GMT. Long-term follow-up is essential for better understanding the clinical features and disease progression in HOD patients, ultimately helping to improve patient outcomes.

## Data Availability

Data are available from the corresponding author on reasonable request.

## Abbreviations

HOD: Hypertrophic olivary degeneration
GMT: Guillain-Mollaret triangle
ION: Inferior olivary nucleus
T2WI: T2-weighted imaging
MRS: Modified rankin scale
ADL: The activities of daily living
MRI: Magnetic resonance imaging
PDWI: Roton density-weighted imaging
tDCS: Transcranial direct current stimulation
TMS: Transcranial magnetic stimulation
